# Predictive Significance of a New Prognostic Score for Patients With Diffuse Large B-Cell Lymphoma in the Interim-Positron Emission Tomography Findings

**DOI:** 10.1097/MD.0000000000002808

**Published:** 2016-02-12

**Authors:** Yu Kong, Lili Qu, Yuekai Li, Dai Liu, Xuemin Lv, Jiankui Han

**Affiliations:** From the Department of Nuclear Medicine, Qilu Hospital of Shandong University, Jinan, Shandong, China.

## Abstract

We hypothesized that the objective treatment response of patients with diffuse large B-cell lymphoma (DLBCL) was affected by many factors such as pathophysiological, biological, and pharmaceutical mechanisms. This retrospective study aimed to evaluate the predictive significance of clinical prognostic factors and interim fluorine-18-fluorodeoxyglucose (^18^F-FDG) positron emission tomography/computed tomography (PET/CT), and to find a new prognostic predictor significantly associated with DLBCL patients’ outcome. A total of 105 adult patients with DLBCL were reviewed. Each patient underwent an interim ^18^F-FDG PET/CT scan after the second chemotherapy cycle. The visual method based on the Deauville 5-point scale was used to evaluate the interim-PET/CT scans. The relationships among the prognostic factors, the 3-year progression-free survival (PFS) rate and overall survival (OS) rate were analyzed with Kaplan–Meier plots. The predictive value of the newly constructed prognostic score was analyzed with multivariate analysis (Cox proportional hazard regression model). The visual analysis showed statistically significant differences in both PFS and OS between the patients with a negative interim-PET/CT and those with a positive interim-PET/CT. Advanced age, advanced stage, and DLBCL subtype were also significantly associated with outcome. A new prognostic score that composed of the above 4 factors was obtained. New prognostic score stratified patients into 4 risk groups with 3-year PFS of 98.5%, 73.9%, 11.1%, and 0%, and 3-year OS of 100%, 91.3%, 55.6%, and 0% (*P* < 0.001 for PFS and OS). Multivariate analysis showed that the new prognostic score had the greatest ability to predict relapse (*P* < 0.001) and death (*P* < 0.001). In DLBCL patients, interim ^18^F-FDG PET/CT can provide significant independent prognostic information. Our work illustrates that the new prognostic score has the strongest potential for accurately prognostication, for stratification in clinical trials, and for design of novel strategies for DLBCL patients in the high-risk group.

## INTRODUCTION

Diffuse large B-cell lymphoma (DLBCL) accounts for approximately 30% of all non-Hodgkin lymphomas (NHLs) diagnoses in adults. Increased morbidity and mortality rates have been reported in recent years. Most DLBCL patients respond well to first-line therapy, but some patients eventually relapse after a complete response (CR) to initial therapy.^1,2^ Therefore, the ability to accurately categorize patients into distinct prognostic groups (with significant outcome differences) is becoming an urgent necessity for clinicians.

A well-established predictor of lymphoma is the International Prognostic Index (IPI),^3,4^ which is built on 5 therapeutic clinical characteristics as following: age, the Ann Arbor stage, Eastern Cooperative Oncology Group (ECOG) performance status, number of extranodal disease, and lactate dehydrogenase (LDH) level. One point is gained for each of the following risk factors: age >60 years; stage III or IV; more than 1 extranodal disease; ECOG performance status of 2, 3, or 4; or serum LDH upper limit normal. The evaluation standard is 0 or 1 (low risk), 2 (low-intermediate risk), 3 (high-intermediate risk), and 4 or 5 (high risk). The IPI has been considered as a significant prognostic predictor which could affect the clinical risk stratification before treatment. However, from another point of view, the value of IPI in predicting the prognosis of lymphoma was limited by various factors. This raises the question of whether IPI is still significant independent predictor in patients with DLBCL, or whether other clinical factors are now more relevant.

Fluorine-18-fluorodeoxyglucose (^18^F-FDG) positron emission tomography/computed tomography (PET/CT), unlike conventional CT, can provide both functional and morphological data. It has been demonstrated that in lymphoma, ^18^F-FDG PET/CT is better for anatomic imaging to assess the viable lesions, initial staging and restaging, treatment response, prognosis, and post-treatment relapse/exacerbation.^5–7^ Response criteria were determined according to the International Workshop Criteria (IWC) 2007 (IWC + PET).^8^ The following PET/CT criteria have been approved to evaluate therapeutic responses: complete response (CR), partial response (PR), stability, and progression/relapse.^9^ In recent decades, interim-PET/CT has been utilized to evaluate the prognosis of lymphoma patients. Due to the diverse performance of PET/CT imaging in DLBCL and the different criteria used to interpret PET/CT, the use of interim-PET/CT alone to evaluate the prognostic value in DLBCL is controversial.

We thought that clinical factors included IPI might reflect the potential response to chemotherapy from the pathophysiological and biological characteristics in the pretreatment status. Moreover, interim-PET/CT could show the changes of tumor metabolic activity and reflect the objective interim treatment response. We hypothesized that patients prognosis was associated with many factors such as pathophysiological, biological, and pharmaceutical mechanisms. Therefore, this retrospective study aimed to analyze the prognostic significance of clinical characteristics together with interim-PET/CT results, and to find a new prognostic predictor significantly associated with DLBCL patients’ outcome.

## MATERIALS AND METHODS

### Patients

Between December 2009 and October 2014, we retrospectively reviewed 105 adult patients newly diagnosed with DLBCL and treated at Qilu Hospital of Shandong University, Jinan, China. The included criteria were as follows: all patients had to have underwent interim whole-body PET/CT after the second cycle of chemotherapy, post-therapy (after initial therapy) whole-body PET/CT, or both, and baseline PET/CT was optional; patients were treated with a regimen of rituximab (R) plus CHOP (cyclophosphamide, doxorubicin, vincristine, and prednisone).^10^ Patients who had primary central nervous system lymphoma, children and adolescents with lymphoma, or interim-PET/CT did not scan after 2 cycles of chemotherapy were excluded. Complete patient histories and relative examination data were collected at each visit.^11,12^ Histopathological specimens and relevant patients cases were reviewed and patients can be classified into germinal center B-cell-like (GCB) and activated B-cell-like (ABC) subtypes by gene-expression profiling (GEP).^13,14^ First-line treatment consisted of 6 to 8 courses of R-CHOP. There were no plans to change the therapy based on the interim-PET/CT results. This study protocol was approved by the ethics committees at Shandong University Cancer Center. All patients provided written informed consent before carrying out the treatment.

### Image Acquisition

^18^F-FDG PET/CT scans from the skull base to the upper thigh were acquired on a Discovery STE16 ultra-highly integrated PET/CT system (GE Healthcare, Milwaukee, WI, USA), which consisted of a spiral CT scanner and a PET scanner; the brain was scanned alone. Patients were fasted for a minimum of 6 h and had blood glucose levels of ≤8.0 mmol/L before being intravenously injected with a weight-based amount of ^18^F-FDG (5.5 MBq/kg). Patients were asked to rest for 40 to 60 min after ^18^F-FDG injection and to void their bladder before image acquisition. First, a whole-body CT scan was performed, with a 16-detector row CT scanner. The following CT image acquisition parameters were used: peak voltage, 120 kV; tube current, 80 mA/s; matrix, 512 × 512; field of view, 50 cm; and slice thickness, 3.75 mm. Immediately after the CT acquisition, PET scans were performed with a 128 × 128 matrix, a 15.7-cm axial field of view, and in 6 to 8 bed positions (150 s acquisition time per bed position). The CT data were used for attenuation correction. PET images were reconstructed iteratively with 3-dimensional ordered subsets expectation maximization (VUE point).

### Interim-PET/CT Interpretation Criteria

An interim-PET/CT scan was generally performed after the second chemotherapy cycle. All PET/CT scans were independently reviewed by at least 2 experienced nuclear medicine physicians using the qualitative method based on the Deauville 5-point scale (PS).^15–17^ The Deauville 5-PS on interim PET analysis was listed as follows: 1, no FDG uptake; 2, FDG uptake ≤ mediastinum; 3, FDG uptake > mediastinum but ≤ liver; 4, FDG uptake moderately increased compared with the liver at any site; 5, FDG uptake markedly increased compared with the liver at any site and new sites and/or new sites of disease. An interim PET result grades 1 to 3 was considered as negative (Deauville score ≤ 3), and an interim PET grades 4 or 5 was considered as positive (Deauville score > 3). The CT image analysis was based on the pathological anatomical features and morphological changes. A negative CT result meant that lymph node and extranodal diseases had disappeared, and a positive CT indicated an abnormal lymph node size and/or pathological anatomical extranodal lesion. The PET/CT results were classified as negative or positive based on the combined PET and CT criteria.

### Statistical Analysis

Progression-free survival (PFS) was defined as the interval between the date of diagnosis and the date of lymphoma progression, first relapse, death from any cause, or the last follow-up date. Overall survival (OS) was deemed to be the time from the date of diagnosis until death from any cause or the last follow-up date. PFS and OS were depicted using Kaplan–Meier plots. Between-group differences were analyzed with the log-rank test. The Cox proportional hazard regression model was used to assess the effects of the relevant prognostic factors on survival times. All statistical analyses were performed using the SPSS 19.0 statistical software package (IBM Corporation, Armonk, NY), and a *P* value of <0.05 was considered to be statistically significant.

## RESULTS

### Patient Characteristics

The clinical characteristics of the 105 patients who met the eligibility criteria are summarized in Table 1. The median age was 56 years (range 19–82 years) with a slight male predominance (54.3%); 40.0% of patients had over 60 years old. Forty-six patients (43.8%) were presented in advanced stage (III/IV). According to GEP, 64 patients were subclassified as GCB DLBCL and 41 patients were ABC DLBCL. According to the final response at the end of first-line therapy, 89 (84.8%) patients achieved CR, 12 (11.4%) patients achieved PR, and 4 (3.8%) patients showed stability or progression. The median patient follow-up was 32 months (range, 9–59 months). Eighty patients showed no relapse (PFS, 76.2%). At the end of the follow-up, the OS was 83.8% (88 patients).

**TABLE 1:**
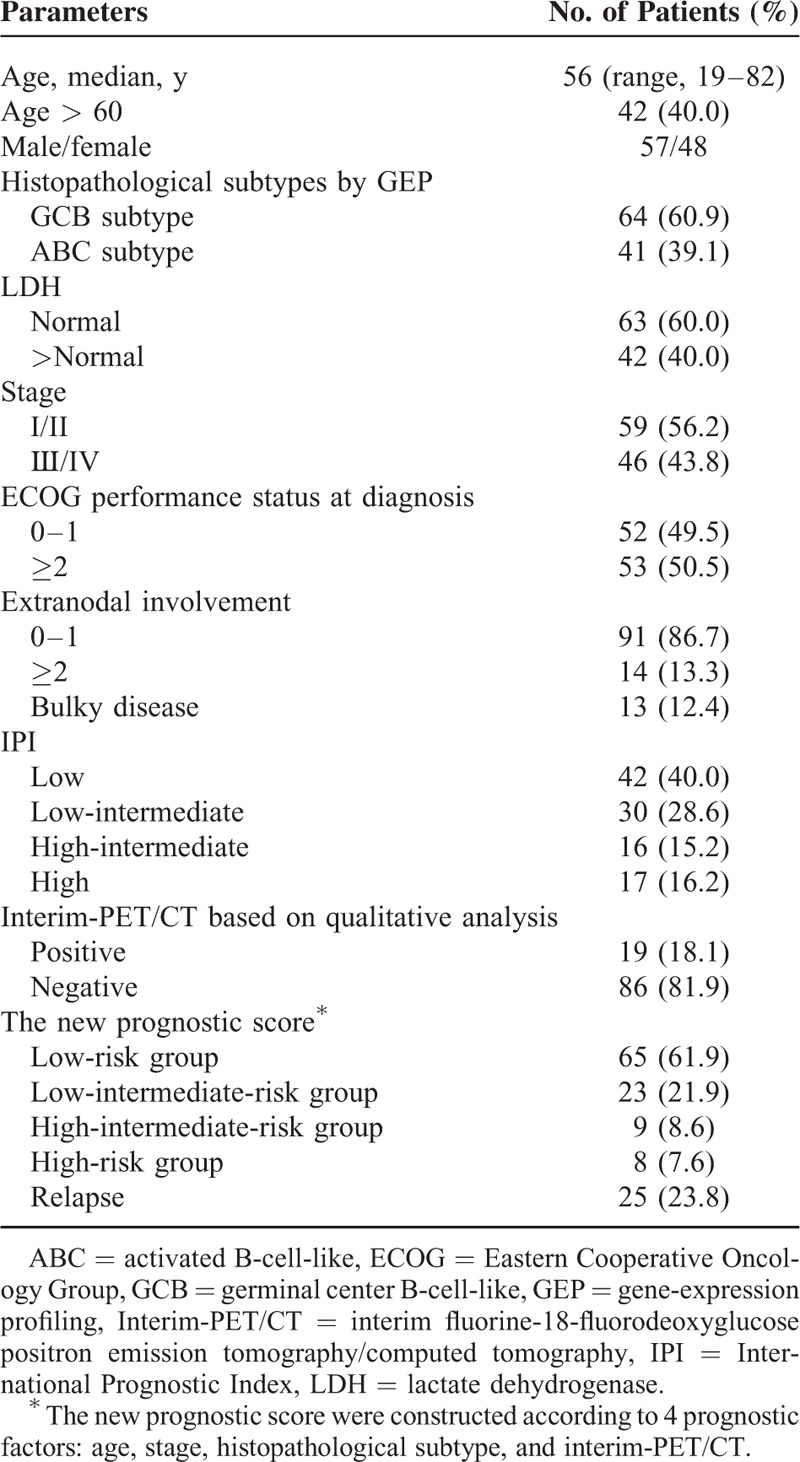
Characteristics of All Diffuse Large B-Cell Lymphoma Patients

### Three-Year PFS and OS Rates According to Interim-PET/CT Results, and Prognostic Factors for DLBCL Patients

**FIGURE 1:**
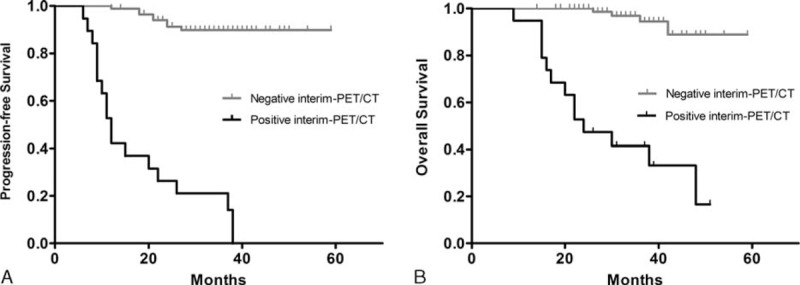
Progression-free survival and overall survival rates of DLBCL patients, according to visual analysis (A, B). DLBCL = diffuse large B-cell lymphoma.

Among the 105 patients, 19 (18.1%) had a positive interim-PET/CT after the second cycle of chemotherapy, while the other 86 patients (81.9%) had a negative interim-PET/CT. The 3-year PFS rates in patients with positive and negative interim-PET/CT were 21.1% and 90.7%, respectively (*P* < 0.001; Table 2, Figure 1A). Qualitative analysis demonstrated the difference in 3-year PFS was statistically significant between the patients with positive interim-PET/CT and the patients with negative interim-PET/CT (*P* < 0.001). Patients who are demonstrating with a negative interim-PET/CT had a significantly lower recurrence rate than the patients with a positive interim-PET/CT. Univariate analysis of PFS in DLBCL patients showed age, ECOG, extranodal involvement, stage, bulky disease, histopathological subtype, and interim-PET/CT had the ability to predict relapse. However, in the multivariable analysis, the following factors were significant for PFS: interim-PET/CT (hazard ratio [HR] 27.565 [8.842–85.938], *P* < 0.001), stage (HR 21.135 [4.088–109.263], *P* < 0.001), histopathological subtype (HR 8.088 [2.707–24.162], *P* < 0.001), and age (HR 5.045 [1.704–14.940], *P* = 0.003).

**TABLE 2:**
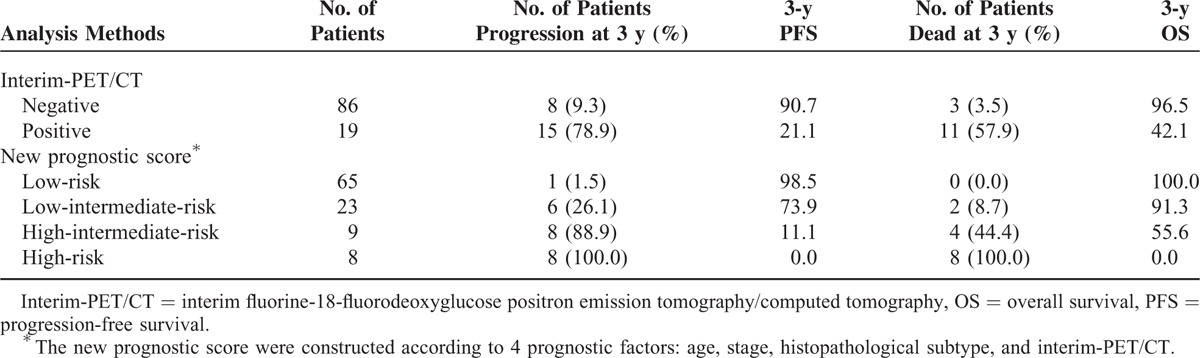
Three-Year PFS and OS According to PET/CT Results and New Prognostic Score

Analysis of the qualitative results showed statistically significant differences in the 3-year OS rates of patients with negative interim-PET/CT (96.5%) and those with positive interim-PET/CT (42.1%) (*P* < 0.001; Table 2, Figure 1B). Patients with a negative interim-PET/CT had a better outcome than patients with a positive interim-PET/CT. Univariate analysis of OS in DLBCL patients showed age, ECOG, extranodal involvement, stage, bulky disease, histopathological subtype, and interim-PET/CT had the ability to predict OS. However, multivariate analysis demonstrated that interim-PET/CT had the greatest ability to predict death (HR 11.373 [3.101–41.708], *P* < 0.001), followed by stage (HR 8.498 [1.008–71.612], *P* = 0.049), age (HR 6.825 [1.573–29.618], *P* = 0.010), and histopathological subtype (HR 5.786 [1.682–19.904], *P* = 0.005). The same 4 factors were also significant for OS. Univariate and multivariate analyses of PFS and OS in DLBCL patients are summarized in Table 3.

**TABLE 3:**
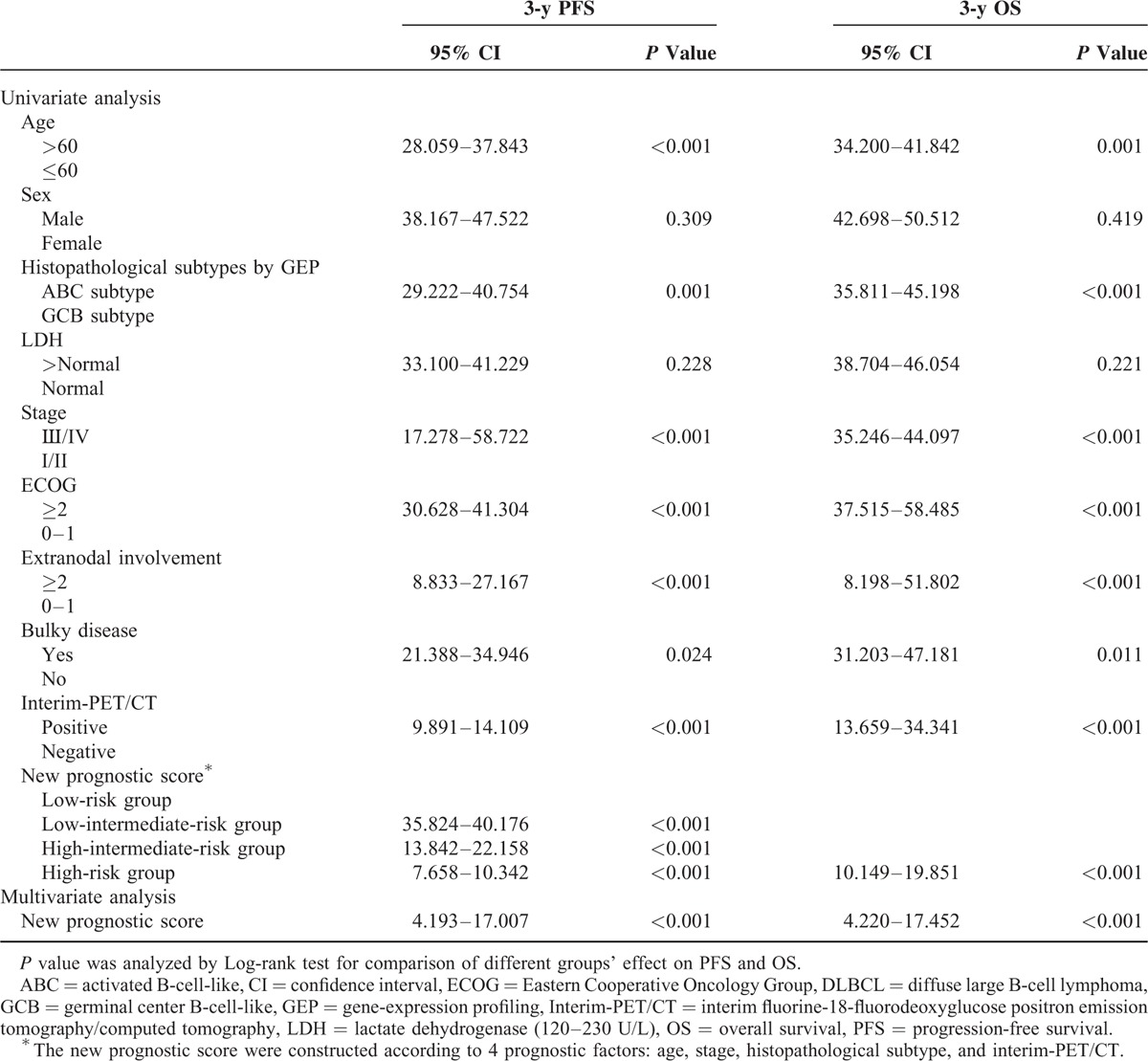
Univariate and Multivariate Analyses of PFS and OS for DLBCL Patients

### New Prognostic Score for DLBCL Patients

**FIGURE 2:**
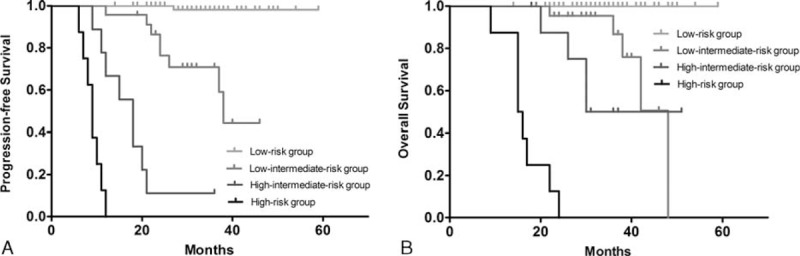
Progression-free survival and overall survival rates of DLBCL patients, according to the new prognostic score (A, B). DLBCL = diffuse large B-cell lymphoma.

Based on the above, we constructed a new prognostic score according to the sum of prognostic factors among positive interim-PET/CT (1 point), age >60 (1 point), stage III or IV (1 point), and ABC subtype (1 point). Patients in the low-risk group (0–1 points) had a 3-year PFS of 98.5% and 3-year OS of 100%, compared to 73.9% and 91.3% for patients in the low-intermediate-risk group (2 points), 11.1% and 55.6% for patients in the high-intermediate-risk group (3 points), and 0% and 0% for patients in the high-risk group (4 points) (*P* < 0.001 for both PFS and OS; Table 2, Figure 2). Multivariate analysis (added new prognostic score into analysis) demonstrated that this new prognostic score was the most significant independent prognostic factor, which had the greatest ability to predict relapse (HR 8.444 [4.193–17.007], *P* < 0.001) and death (HR 8.582 [4.220–17.452], *P* < 0.001; Table 3).

## DISCUSSION AND CONCLUSIONS

In recent years, great effort has been made to improve the prognosis of DLBCL; however, the most effective treatment plan and prognostic evaluation criteria for DLBCL are still undetermined. During the past decade, the 2 most important developments in the treatment of DLBCL have been the use of rituximab and the application of functional imaging in disease staging.^18^^18^F-FDG PET/CT, as 1 functional imaging technique, not only can indicate the size and shape of the lesion, but also can show the metabolic activity of tumors. It allows treatment responses to be assessed earlier and with a greater degree of accuracy than CT. If interim-PET/CT was able to predict the patients who are less likely to be cured by first-line treatment, their therapy could be changed earlier. Meanwhile, patients who respond well to treatment may remain on their original therapy or reduce their therapy cycles. We evaluated the predictive value of interim-PET/CT in predicting relapse and death of patients with DLBCL, and determined which clinical factors affect prognostic significance in those patients. Our study confirmed the prognostic importance of interim-PET/CT for both PFS and OS. The difference in outcome between positive interim-PET/CT and negative interim-PET/CT patients is significantly large in our cohort. Moreover, clinical factors (stage, age, and histopathological subtype) also showed to be important prognostic factors in our study.

**FIGURE 3:**
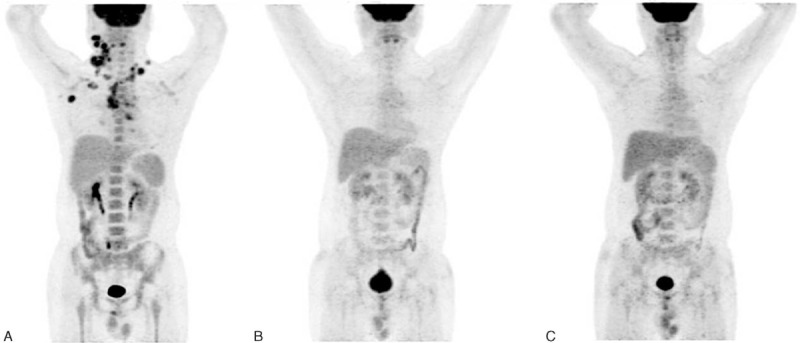
Serial PET images of 30-y-old man with stage II GCB DLBCL, referred for PET/CT for evaluation after 2 cycles of chemotherapy. Figure (A) obtained before chemotherapy shows lymphomatous right neck, bilateral supraclavicular, mediastinal, and right axillary lymph nodes. Figure (B) obtained after 2 cycles of therapy shows no residual abnormal ^18^F-FDG uptake, interpreted as negative interim-PET according to Deauville 5-PS and assigned to low-risk group according to the new prognostic score. Figure (C) obtained after first-line chemotherapy always shows complete remission. ^18^F-FDG = fluorine-18-fluorodeoxyglucose, CT = computed tomography, DLBCL= diffuse large B-cell lymphoma, GCB = germinal center B-cell-like, PET = positron emission tomography, PS = point scale.

Previous studies have suggested that the extensive use of ^18^F-FDG PET/CT has dramatically improved the prognostic evaluation and risk-based stratified treatment of lymphomas.^19^ Lin et al^20^ reported that early pretreatment ^18^F-FDG PET was weakly associated with PFS in DLBCL patients. Chihara et al^21^ observed an association between high SUV_max_ on FDG-PET in the primary diagnosis and shorter OS in patients with DLBCL. Numerous studies have demonstrated that ^18^F-FDG PET/CT at the end of treatment is highly predictive of PFS and OS in aggressive NHL. In recent years, the predictive value of interim ^18^F-FDG PET/CT has been investigated, and the conclusions have varied. Many centers have researched the predictive value of interim-PET/CT in different periods (from after 1 cycle of chemotherapy to the end of 4 cycles of chemotherapy). Interim-PET/CT after 1 cycle of chemotherapy had greater predictive accuracy for PFS compared to post-treatment PET/CT,^22^ whereas the predictive value of interim-PET/CT examined between 2 and 4 cycles varies greatly.^23^ Our study performed interim-PET/CT at the end of 2 cycles of chemotherapy (Figure 3). Different methods for interim-PET/CT analysis (qualitative or semi-quantitative analysis) have led to different predictive consequences. Fuertes et al evaluated 3 methods for interim-PET/CT analysis: qualitative 3-PS, 5-PS, and the semi-quantitative ΔSUV_max_. The authors supported the use of both 5-PS and ΔSUV_max_ for interpreting interim-PET/CT to predict the PFS and OS of DLBCL patients.^24^ Some studies considered that semi-quantitative analysis according to ΔSUV_max_ may be more objective. However, it has not been validated in large multicentre trials. It has not been used here due to the incomplete baseline pretherapy PET/CT imaging. Meanwhile, Schot et al^25^ showed that careful visual assessment of serial PET provides equivalent results to those obtained from measurement of SUV of the most intense pathologic lesions. Interim-PET/CT based on qualitative 5-PS method plays its prognostic importance in our study, and positive interim-PET/CT is 1 of the influential factors which affects the prognosis of patients with DLBCL.

**FIGURE 4:**
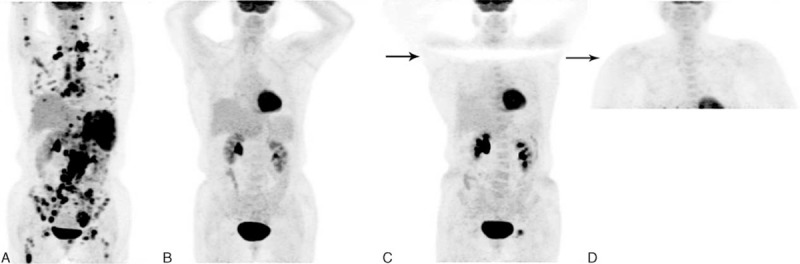
Serial PET images of 80-y-old man with stage IV GCB DLBCL, referred for PET/CT for evaluation after 2 cycles of chemotherapy. Figure (A) shows lesions status before chemotherapy (many regional lymph nodes and multiple organs involvement). Figure (B) obtained after 2 cycles of therapy shows no obvious residual abnormal ^18^F-FDG uptake, interpreted as negative interim-PET according to Deauville 5-PS and assigned to low-intermediate-risk group according to the new prognostic score. After first-line chemotherapy, patient achieves complete remission. Figure (C) obtained 14 mo later, 1 left inguinal lymph node enlarges and shows abnormal increased ^18^F-FDG uptake, which means the disease progresses. Because the patient moves during PET/CT scanning, we rescan the neck and chest imaging (D), and the corresponding area shows no lesions. ^18^F-FDG = fluorine-18-fluorodeoxyglucose, CT = computed tomography, DLBCL= diffuse large B-cell lymphoma, GCB = germinal center B-cell-like, PET = positron emission tomography, PS = point scale.

In our analysis, the prognostic significance of many clinical factors appears to be surpassed by the interim-PET/CT. However, we found that age and stage which among IPI 5 clinical characteristics and DLBCL subtypes were still associated with patients’ outcome by multivariate analysis when interim-PET/CT was considered to be a prognostic predictor (Figure 4). Many previous reports have demonstrated the predictive significance of these 3 factors for DLBCL patients after front-line therapy.^26,27^ Meanwhile, we note that patients with advanced age, advanced stage, or in ABC group were in large proportion in the positive interim-PET/CT population, and showed a poorer outcome.

**FIGURE 5:**
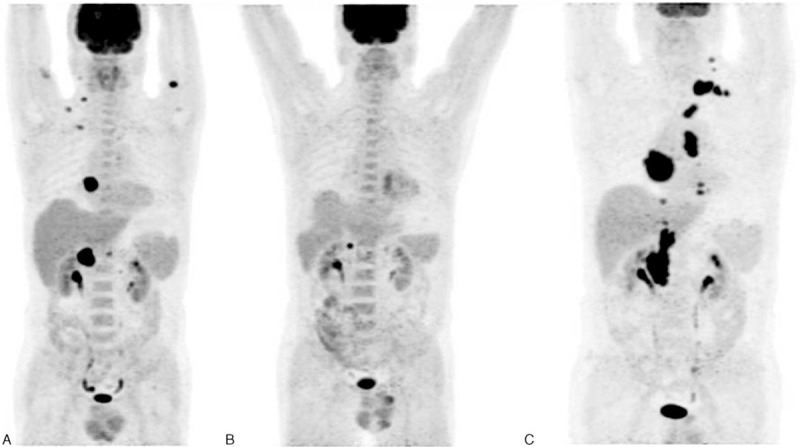
Serial PET images of 32-y-old man with stage IV ABC DLBCL, referred for PET/CT for evaluation after 2 cycles of chemotherapy. Figure (A) shows lesions status before chemotherapy. Figure (B) obtained after 2 cycles of therapy shows the positive uptake at residual right adrenal gland and the focal uptake at lumbar vertebral, interpreted as positive interim-PET according to Deauville 5-PS and assigned to high-intermediate-risk group according to the new prognostic score. Figure (C) obtained 3 mo later after first-line chemotherapy and prompted disease progression (lesions expanded and increased). ABC = activated B-cell-like, CT = computed tomography, DLBCL= diffuse large B-cell lymphoma, PET = positron emission tomography, PS = point scale.

**FIGURE 6:**
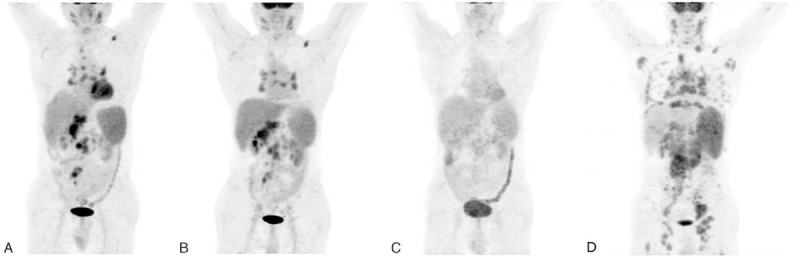
Serial PET images of 68-y-old man with stage IV ABC DLBCL, referred for PET/CT for evaluation after 2 cycles of chemotherapy. Figure (A) shows lesions status before chemotherapy. Figure (B) obtained after 2 cycles of therapy shows the positive uptake at residual left supraclavicular, mediastinal, abdominal, and retroperitoneal lymph nodes, interpreted as positive interim-PET according to Deauville 5-PS and assigned to high-risk group according to the new prognostic score. Figure (C) obtained after first-line chemotherapy shows no obvious uptake at any sites. However, the disease relapses 3 mo later, and widely spreads to many regional lymph nodes, spleen, lungs, and bones (D). ABC = activated B-cell-like, CT = computed tomography, DLBCL= diffuse large B-cell lymphoma, PET = positron emission tomography, PS = point scale.

The new prognostic score in our present study is simple to calculate for clinicians and has high stratification and prognostication ability, with 3-year PFS ranging from 98.5% to 0% and 3-year OS from 100% to 0%. Our prognostic score includes 4 factors: age, stage, histopathological subtype, and interim-PET/CT. Age and stage maybe reflect the potential response to chemotherapy from the physiological and biological characteristics in the pretreatment status. Histopathological subtypes reflect the initial state of DLBCL from molecular biology and genetic heterogeneity level. Interim-PET/CT shows the changes of tumor metabolic activity and reflects the objective interim treatment response during treatment. The new score has divided patients into 4 groups: low-risk group, low-intermediate-risk group, high-intermediate-risk group (Figure 5), and high-risk group, further risk stratified patients with DLBCL. The most important potential use of this score was to identify high-risk patients whose outcomes after first-line therapy are dismal (Figure 6). Based on these results, we can strongly suggest that such patients should be considered for other therapy strategies or for prospective clinical trials testing new therapeutic approaches. Patients in low-risk group were considered to complete their initial first-line treatment and regularly follow-up. Patients in low-intermediate-risk group or high-intermediate-risk group were considered to increase chemotherapy cycles/dose or change treatment strategies. We should be looking for evaluating the prognosis of patients in intermediate-risk group in the future research, further improve their OS rates.

The present study contains a number of limitations because of its retrospective nature. Treatment and testing protocols were not prespecified. Consequently, there might be variation among the institutions. These issues should be addressed in future research.

As showed above, our results demonstrate the clinical factors, including advanced age, advanced stage, ABC subtype, and positive interim-PET/CT, that could predict the prognosis of DLBCL patients. The new predictive score based on the above 4 factors, which proved as the most significant independent predictor can improve clinicians ability to risk stratify patients with DLBCL, and to design numerous appropriate and sensitive therapeutic approaches. Larger and prospective researches are warranted to confirm these above results.

## References

[1] Chao MP Treatment challenges in the management of relapsed or refractory non-Hodgkin's lymphoma—novel and emerging therapies. *Cancer Manag Res* 2013; 5:251–269. 24049458 10.2147/CMAR.S34273 PMC3775637

[2] Kewalramani T Zelenetz AD Nimer SD Rituximab and ICE as second-line therapy before autologous stem cell transplantation for relapsed or primary refractory diffuse large B-cell lymphoma. *Blood* 2004; 103:3684–3688. 14739217 10.1182/blood-2003-11-3911

[3] Haioun C Itti E Rahmouni A [18F] fluoro-2-deoxy-d-glucose positron emission tomography (FDG-PET) in aggressive lymphoma: an early prognostic tool for predicting patient outcome. *Blood* 2005; 106:1376–1381. 15860666 10.1182/blood-2005-01-0272

[4] Shipp MA Harrington PD A predictive model for aggressive non-Hodgkin's lymphoma. The International Non-Hodgkin's Lymphoma Prognostic Factors Project. *N Engl J Med* 1993; 329:987–994. 8141877 10.1056/NEJM199309303291402

[5] Araf S Montoto S The use of interim ^18^F-fluorodeoxyglucose PET to guide therapy in lymphoma. *Future Oncol* 2013; 9:807–815. 23718301 10.2217/fon.13.55

[6] Cortés Romera M Gámez Cenzano C Caresia Aróztegui A Utility of the PET–CT in the evaluation of early response to treatment in the diffuse large B-cell lymphoma. Preliminary results. *Rev Esp Med Nucl Ima Mol (English Edition)* 2012; 31:135–141. 10.1016/j.remn.2011.05.011 21944191

[7] Tateishi U PET/CT in malignant lymphoma: basic information, clinical application, and proposal. *Int J Hematol* 2013; 98:398–405. 24065513 10.1007/s12185-013-1444-3

[8] Cheson BD Pfistner B Juweid ME Revised response criteria for malignant lymphoma. *J Clin Oncol* 2007; 25:579–586. 17242396 10.1200/JCO.2006.09.2403

[9] Dupas B Augeul-Meunier K Frampas E Staging and monitoring in the treatment of lymphomas. *Diagn Interv Imaging* 2013; 94:145–157. 23332618 10.1016/j.diii.2012.12.009

[10] Pfreundschuh M Trümper L Österborg A CHOP-like chemotherapy plus rituximab versus CHOP-like chemotherapy alone in young patients with good-prognosis diffuse large-B-cell lymphoma: a randomised controlled trial by the MabThera International Trial (MInT) Group. *Lancet Oncol* 2006; 7:379–391. 16648042 10.1016/S1470-2045(06)70664-7

[11] Dorth JA Prosnitz LR Broadwater G Radiotherapy dose–response analysis for diffuse large B-cell lymphoma with a complete response to chemotherapy. *Radiat Oncol* 2012; 7:100. 22720801 10.1186/1748-717X-7-100 PMC3464871

[12] Yoo C Lee DH Kim JE Limited role of interim PET/CT in patients with diffuse large B-cell lymphoma treated with R-CHOP. *Ann Hematol* 2011; 90:797–802. 21181163 10.1007/s00277-010-1135-6

[13] Alizadeh AA Eisen MB Davis RE Distinct types of diffuse large B-cell lymphoma identified by gene expression profiling. *Nature* 2000; 403:503–511. 10676951 10.1038/35000501

[14] Wright G Tan B Rosenwald A A gene expression-based method to diagnose clinically distinct subgroups of diffuse large B cell lymphoma. *Proc Natl Acad Sci USA* 2003; 100:9991–9996. 12900505 10.1073/pnas.1732008100 PMC187912

[15] Itti E Meignan M Berriolo-Riedinger A *Eur J Nucl Med Mol Imaging* 2013; 40:1312–1320. 23649463 10.1007/s00259-013-2435-6

[16] Moskowitz CH Interim PET-CT in the management of diffuse large B-cell lymphoma. *ASH Educ Program Book* 2012; 2012:397–401. 10.1182/asheducation-2012.1.397 23233610

[17] Meignan M Gallamini A Meignan M Report on the First International Workshop on Interim-PET-Scan in Lymphoma. *Leuk Lymphoma* 2009; 50:1257–1260. 19544140 10.1080/10428190903040048

[18] Alousi AM Saliba RM Okoroji GJ Disease staging with positron emission tomography or gallium scanning and use of rituximab predict outcome for patients with diffuse large B-cell lymphoma treated with autologous stem cell transplantation. *Br J Haematol* 2008; 142:786–792. 18564354 10.1111/j.1365-2141.2008.07277.x

[19] Bai B Huang H-Q Cai Q-C Predictive value of pretreatment positron emission tomography/computed tomography in patients with newly diagnosed extranodal natural killer/T-cell lymphoma. *Med Oncol* 2013; 30:1–10. 10.1007/s12032-012-0339-0 23329306

[20] Lin C Itti E Haioun C Early ^18^F-FDG PET for prediction of prognosis in patients with diffuse large B-cell lymphoma: SUV-based assessment versus visual analysis. *J Nucl Med* 2007; 48:1626–1632. 17873129 10.2967/jnumed.107.042093

[21] Chihara D Oki Y Onoda H High maximum standard uptake value (SUV_max_) on PET scan is associated with shorter survival in patients with diffuse large B cell lymphoma. *Int J Hematol* 2011; 93:502–508. 21512731 10.1007/s12185-011-0822-y

[22] Kostakoglu L Coleman M Leonard JP PET predicts prognosis after 1 cycle of chemotherapy in aggressive lymphoma and Hodgkin's disease. *J Nucl Med* 2002; 43:1018–1027. 12163626

[23] Mikhaeel N Hutchings M Fields P FDG-PET after two to three cycles of chemotherapy predicts progression-free and overall survival in high-grade non-Hodgkin lymphoma. *Ann Oncol* 2005; 16:1514–1523. 15980161 10.1093/annonc/mdi272

[24] Fuertes S Setoain X Lopez-Guillermo A Interim FDG PET/CT as a prognostic factor in diffuse large B-cell lymphoma. *Eur J Nucl Med Mol Imaging* 2013; 40:496–504. 23344136 10.1007/s00259-012-2320-8

[25] Schot BW Pruim J van Imhoff GW The role of serial pre-transplantation positron emission tomography in predicting progressive disease in relapsed lymphoma. *Haematologica* 2006; 91:490–495. 16533726

[26] Rosenwald A Wright G Chan WC The use of molecular profiling to predict survival after chemotherapy for diffuse large-B-cell lymphoma. *N Engl J Med* 2002; 346:1937–1947. 12075054 10.1056/NEJMoa012914

[27] Lanic H Mareschal S Mechken F Interim positron emission tomography scan associated with International Prognostic Index and germinal center B cell-like signature as prognostic index in diffuse large B-cell lymphoma. *Leuk Lymphoma* 2012; 53:34–42. 21806349 10.3109/10428194.2011.600482

